# Chemists Forge Paths to Inaccessible Sugars

**DOI:** 10.1021/acscentsci.3c01371

**Published:** 2023-11-21

**Authors:** XiaoZhi Lim

When Clay Bennett started his postdoctoral stint in 2005, he thought—“naively,” he admits—that he would spend just a couple of months preparing a few grams of an oligosaccharide before using the molecule in his actual research project studying enzyme activity.

The synthesis wouldn’t take long, he predicted, because an oligosaccharide is just a few sugar molecules strung together: a chain of rings containing carbon atoms and an oxygen, decorated with simple hydroxyl groups on the periphery.

But a year later, after watching colleagues rack up publications, Bennett was still struggling to make enough of the oligosaccharide. He eventually abandoned his project. “It was just untenable,” Bennett recalls. The oligosaccharide “was just the tool, and I realized that the tool was a project in itself.”

What Bennett—now a carbohydrate chemist at Tufts University—experienced is common among those investigating sugar molecules. Carbohydrates are ubiquitous, occurring on the surfaces of proteins and cells, where they are crucial for protein folding as well as biomolecular and cellular interactions. Many biologically active natural products also carry sugar motifs, making carbohydrates vitally important for therapeutics.

**Figure:**
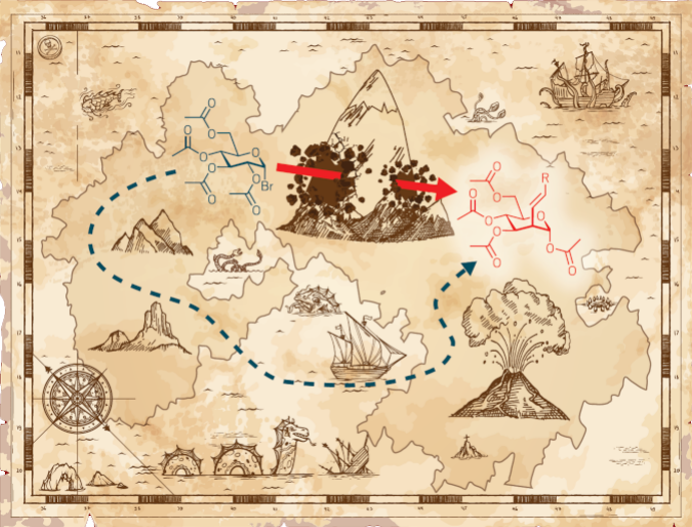
Credit: Shutterstock/Yang H. Ku/C&EN.

To unlock the potential of sugars, scientists need to study them, and studying sugars means preparing enough of them in the lab. But because carbohydrates sport multiple, virtually identical hydroxyl groups and daunting stereochemistry, the molecules are among the most challenging to synthesize.

“If you want complex sugars, there’s a handful of things that are relatively easy to access,” Bennett says. “But anything else is going to be a slog.

Bennett and other chemists want to ease the pain of sugar synthesis by adapting ever-more-precise reactions and methods to easily invert specific stereocenters or swap functional groups while leaving the rest of the molecule untouched. These structural transformations could help biologists and medicinal chemists gain access to rare sugars, allowing scientists to study the molecules’ biological roles or to tweak natural products to develop new drugs. “I think it’s the best time to be a sugar researcher,” says Steven D. Townsend, a carbohydrate scientist at Vanderbilt University.

## More than calories

Nonbiochemists often associate carbohydrates with food, but sugar biochemistry boasts far more complex structures and functions than the molecules in bread or pasta do. The carbohydrates achieve stunning diversity by varying the positions of hydroxyl, methyl, and other groups tacked on to 3D rings and by changing how those rings themselves are linked together.

When attached to small molecules, sugars often control key properties. “If you remove the carbohydrate units from the natural product, you actually will remove its biological activities,” says Ming-Yu Ngai, an organic chemist at Purdue University.

Sugar molecules can also help cells recognize one another. Pathogens often take advantage of this system to infect cells, as scientists saw during the COVID-19 pandemic, Ngai says. The SARS-CoV-2 coronavirus is covered in saccharides that it uses to bind to and infiltrate human cells. Understanding the role that sugars play in viral or bacterial infections could lead to novel carbohydrate-based therapeutics.

For instance, a cross-institutional team showed that cyanovirin-N, a carbohydrate-binding protein isolated from blue-green algae, can block SARS-CoV-2 from infecting cells. The protein works by glomming on to the oligosaccharides covering the virus’s spike proteins. Cyanovirin-N could offer broad protection against different variants. “As SARS-CoV-2 has mutated, it has not become less sensitive to cyanovirin-N,” says Barry O’Keefe, a senior scientist at the U.S. National Cancer Institute and an author on the study. “In fact, it has become more sensitive,” he says, as the virus adds sugars in its effort to evade our immune system.

**Figure:**
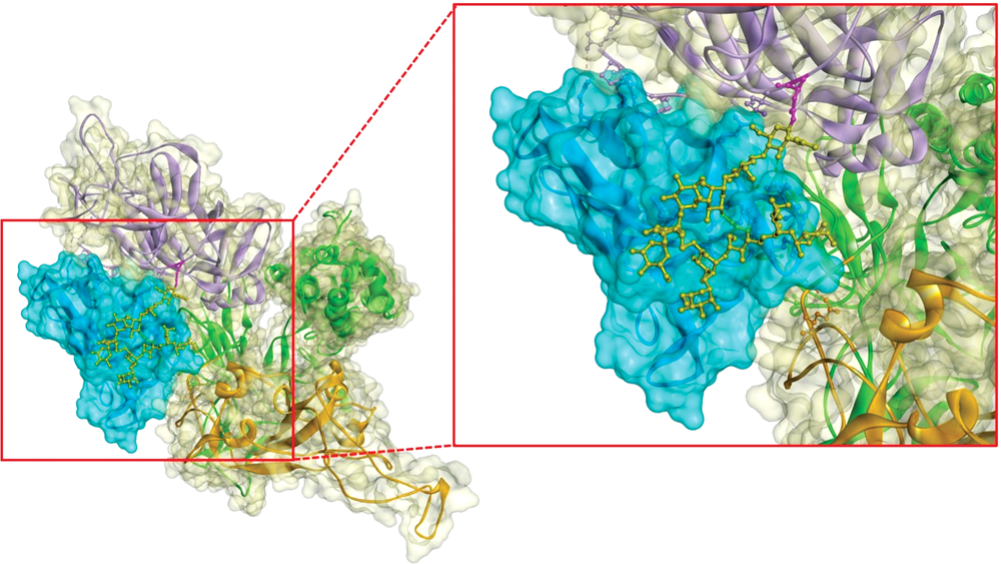
Cyanovirin-N (blue) can grab on to an oligosaccharide (ball-and-stick structure) on the SARS-CoV-2 spike protein, hindering the virus’s ability to infect cells. Credit: Gemma Villorbina.

Sugars themselves also could be the key to new therapies. For example, doxorubicin is an antibiotic and a chemotherapeutic, but the drug’s use is limited because it causes deadly heart attacks at high doses. In 2021, Townsend’s team reported that replacing the monosaccharide on doxorubicin with synthetic sugars changes the drug’s mechanism of cytotoxicity. The modified doxorubicin molecules not only exhibited fewer cardiac side effects but also increased its cancer-killing capabilities.

Townsend’s group is now studying erythropoietin, a protein that stimulates red blood cell production in the bone marrow and is used to treat anemia. Researchers have shown that tweaks to the thicket of oligosaccharides decorating erythropoietin can boost its activity. For example, in 2020, researchers at Osaka University reported that a version of the protein with an extra chain of three sugars boosted red blood cell count in mice to a level comparable to what was achieved with commercial erythropoietin but at less than one-third the dose.

### Hard to get

To obtain sugars for their erythropoietin experiments, Townsend’s lab members found themselves combing through an unexpected substance—egg yolks. Inside these golden packages was a complex sugar that gave the researchers a head start in their synthesis. “We’re a little bit lucky,” Townsend says, because they can now begin their synthesis from a relatively accessible molecule.

Unlike abundant food or biomass sugars such as glucose, many saccharides often occur in tiny quantities and in mixtures that are hard or impossible to separate. For example, among some 2,000 oligosaccharides that occur within human milk, just 5 can be purified in large enough quantities for research, Townsend says.

When chemists can’t obtain a rare sugar from a natural source, they try extracting easier-to-source sugars and then modifying them, as Townsend’s group does with the egg yolk sugars. But this strategy can be tricky. Often, the modifications needed to create the desired sugar involve targeting the hydroxyl groups on the sugar rings. Changing a specific hydroxyl without touching the others is a synthetic challenge.

To distinguish one hydroxyl group from another, carbohydrate chemists have developed an elaborate system of protecting them—for instance, converting certain hydroxyl groups temporarily to ethers such that they become unreactive while leaving other hydroxyls available for reaction. Once the chemists have modified their desired hydroxyl, they can remove the protection on the others.

“If you read a modern oligosaccharide synthesis paper, most of the steps are still protection–deprotection kinds of steps,” says Mark S. Taylor, an organic chemist at the University of Toronto.

“It’s hugely wasteful,” says Alison Wendlandt, an organic chemist at the Massachusetts Institute of Technology. Protection and deprotection don’t just add extra synthetic steps; choosing which protecting groups to use and in what order is an entire area of study.

But selectively modifying hydroxyls isn’t the only difficult part of sugar synthesis. Synthetic chemists also need to wrestle with stereochemistry of various kinds. For one, a monosaccharide’s identity and function depend largely on whether each of the four or five groups sticking off its ring is in an equatorial or axial position. Then, when building chains of sugars, chemists need to form glycosidic bonds—the linkages connecting sugar units in an oligosaccharide—in the correct of two possible configurations. If any of these stereocenters or glycosidic bonds are installed using a reaction that doesn’t select for a single isomer, the result will be a pair of isomers, and the amount of usable product halves.

As a result, chemists often don’t have much material to show for these awkward syntheses. “There are products where 0.7% overall yield was considered to be a gold standard at one point in time,” Bennett says.

**Figure:**
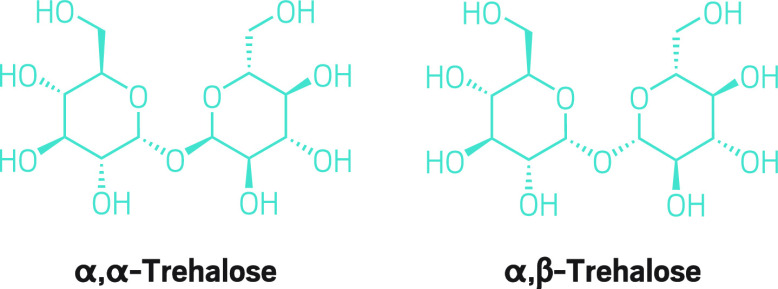
Although trehalose contains just two sugar units, synthesizing it and its derivatives is a challenge because chemists must distinguish between making the α,α and α,β diastereomers. The two structures differ at just one glycosidic bond, but they have different biological activities.

### Radical reactions

To get around these obstacles, the current generation of carbohydrate makers is turning to new catalysts that are so selective that they let chemists skip some protection steps.

At MIT, Wendlandt and her team use radical chemistry to flip the configuration of selected stereocenters in sugar molecules in a single step. On paper, the catalytic reaction looks relatively straightforward—simply swapping a hashed line for a wedge—but it gives chemists access to a world of selectively edited sugars.

The reaction uses blue light and an organic photocatalyst to generate a radical, which removes a hydrogen atom at the C3 position of sugars. A thiol reagent swoops in from the opposite direction and adds back a hydrogen atom, effectively flipping the stereochemistry of the original hydroxyl group from an equatorial to axial placement.

Using this method, Wendlandt’s team converted common α-methylglucose and sucrose into rare sugars such as d-α-methylallose and d-allosucrose, respectively. “We’ve spent way too many steps on them,” Bennett says, recalling his team’s experience synthesizing rare sugars before the new method. “To be able to do it in two or three transformations is absolutely beautiful.” Wendlandt’s group has more recently converted a sugar’s hydroxyl groups in the opposite direction, from axial to equatorial.

**Figure:**
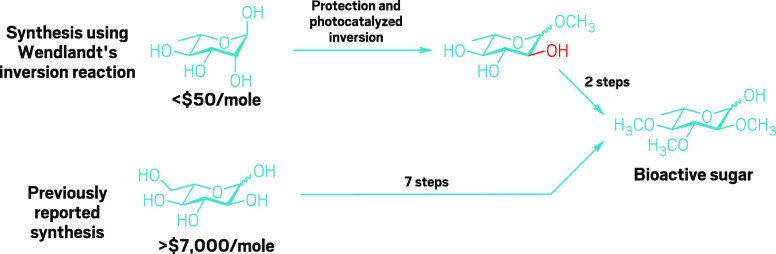
By using an axial-to-equatorial inversion reaction, Alison Wendlandt’s group synthesized a bioactive sugar from cheap l-rhamnose (top) instead of modifying the rare, expensive l-glucose (bottom). The path starting from rhamnose boosts overall yield from 13% to 88%.

Ngai’s team in collaboration with Peng Liu’s research group at the University of Pittsburgh is also using radical catalysis to edit carbohydrates directly. The quick reactions those teams are devising create new carbon–carbon bonds between sugars and larger molecules like pharmaceuticals, producing new leads for drug developers to explore.

The researchers start from sugars with bromide groups on their C1 position, which are easy to prepare, and then add alkene groups to the sugars’ C2 position. The alkene can then serve as a handle for chemists to attach the sugars to lipids, proteins, or drug molecules. Adding the alkene happens in one step, a process that previously would have required eight. Using this reaction, the researchers prepared dozens of edited carbohydrates, including a derivative of ibuprofen.

A major target for some carbohydrate chemists is building sugars that coat the cell membranes of bacteria, particularly antibiotic-resistant ones. Learning how bacteria use those sugars to communicate and form new colonies could lead to new strategies for developing antibiotics.

**Figure:**
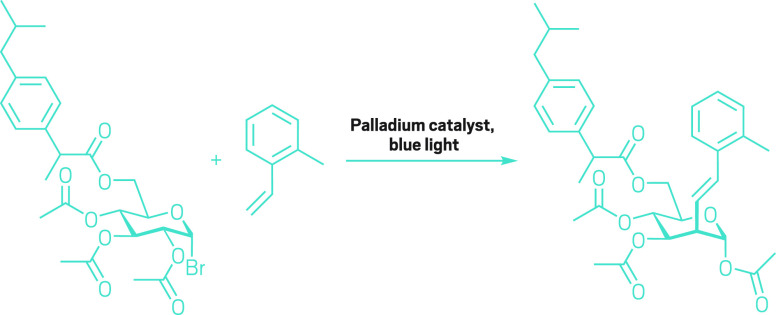
This reaction devised by Ming-Yu Ngai’s and Peng Liu’s groups can easily make sugar-functionalized derivatives of larger molecules, like this modified ibuprofen molecule.

At the University of Bristol, M. Carmen Galan and her team have been working to develop a synthesis for trehalose derivatives, disaccharides found on the bacteria that cause tuberculosis. Although trehalose consists of just two linked glucose units, forming the glycosidic bond between the two in the way the bacteria form it—in the symmetrical α,α conformation rather than in the unsymmetrical α,β one—has been a challenge that has slowed work on the disease.

Using gold catalysts, Galan and her team were able to generate a library of 10 synthetic trehalose derivatives all linked in the way found on bacterial cell walls. What’s more, the reaction produces the same linkage regardless of the stereochemistry of the starting sugars.

Galan and her collaborators are now testing these synthetic analogs as fluorescent tags that could help detect slow-growing tuberculosis bacteria, catching an infection before it becomes fatal.

### Synthetic wish list

At Vanderbilt, Townsend says his lab still leans on sugar synthesis reactions developed in the 1970s and 1980s because they’re highly reliable for preparing hundreds of grams of sugars. What carbohydrate chemists need is not just selective reactions but more reactions that are scalable and easy to use, he says.

A dream in the field is to automate oligosaccharide synthesis, just as peptides and nucleotides can be automatically synthesized in bulk, Bennett says. Several such machines have emerged since Peter H. Seeberger’s team, then at the Massachusetts Institute of Technology, retooled a peptide synthesizer and reported its first automated system in 2001, but all these systems are still limited in scope and can struggle to control the stereochemistry with certain glycosidic bonds. Researchers could make many more improvements, Bennett says.

Bennett’s lab is working on the automated synthesis of oligosaccharides carrying so-called orthogonally protected building blocks—sugars with multiple protecting groups that can be removed one by one using different chemical conditions. These building blocks could be quickly linked into long carbohydrate molecules without risking having the wrong hydroxyl group react.

In collaboration with Nicola L. B. Pohl of Indiana University Bloomington, Bennett’s team developed a continuous-flow system controlled with an open-source program called MechWolf, which can execute a sequence of protection reactions that the user inputs. “You could set it up in the background and let it run while you do something more interesting,” Bennett says. Using this method, the researchers prepared a variety of deoxygenated sugars—bacterial carbohydrates that are missing one of the typical hydroxyl groups—and protected them in just a few hours. Before, such work could have taken a week or more, Bennett says.

Galan thinks that putting protected sugar building blocks at chemists’ fingertips would be a game changer, whether they are products of an automated system like that of Bennett and Pohl or from a commercial chemical supplier. Such products could make carbohydrate chemistry less daunting and more inviting to noncarbohydrate chemists, she says.

On the other hand, for chemists like Wendlandt, the sheer challenge of figuring out better ways to manipulate sugars is the main draw. “Usually when something is really hard, it’s a good sign that it’s a problem worth tackling.”

## XiaoZhi Lim is a freelance contributor to

Chemical & Engineering News, *the independent news outlet of the American Chemical Society*.

